# Oxalate synthesis pathways that contribute to oxalate accumulation in leaves of bitter dock (*Rumex obtusifolius* L.) differ between day and night

**DOI:** 10.5511/plantbiotechnology.25.1124a

**Published:** 2026-03-25

**Authors:** Wakana Sakuma, Hideki Murayama, Atsuko Miyagi

**Affiliations:** 1 Graduate School of Agriculture, Yamagata University, 1-23 Wakaba-machi, Tsuruoka, Yamagata 997-8555, Japan; 2 Faculty of Agriculture, Yamagata University, 1-23 Wakaba-machi, Tsuruoka, Yamagata 997-8555, Japan

**Keywords:** CE-MS/MS, isocitrate pathway, itaconate, oxalate, *Rumex obtusifolius*

## Abstract

Oxalate is a simple dicarboxylate that accumulates primarily in terrestrial parts of plants. Inhibition experiments of a previous study focusing on “new leaves” of *Rumex obtusifolius* revealed that the isocitrate pathway, one of three oxalate pathways, is the primary contributor to oxalate accumulation. However, that experiment was conducted in the dark. In the present study, we conducted inhibitor experiments to evaluate the contribution of the isocitrate pathway to oxalate accumulation in the light. Oxalate accumulation was not inhibited by itaconate in the light, but uptake of itaconate in leaves of *R. obtusifolius* was greater in the light than in the dark. Multivariate statistical analyses revealed that itaconate only slightly affected the metabolite content under light conditions, but itaconate significantly affected the contents of oxalate and related compounds in the dark. These data suggest that the oxalate synthesis pathways that contribute to oxalate accumulation differ between day and night.

## Introduction

*Rumex obtusifolius* L. (Polygonaceae), a perennial herbaceous weed native to Europe, grows well in nutrient-rich soils such as grasslands, fields, and meadows. As the biomass of *R. obtusifolius* increases, the growth of surrounding crops and pasture plants is inhibited (Holm et al. 1977). Furthermore, *R. obtusifolius* exhibits high tolerance to adverse environmental conditions, such as acidic soils, aluminum ion stress, and low temperature (Horie and Nemoto 1990; Miyagi et al. 2010a, 2013c). *R. obtusifolius* also produces a large number of seeds, which have a high germination rate (Cavers and Harper 1964). Eradicating *R. obtusifolius* is difficult because the plants can produce new leaves from underground rhizomes even after the terrestrial portions of the plant are cut, with the rhizomes providing nutrition for reproduction (Cavers and Harper 1964; Hongo 1989; Pino et al. 1995). It has been reported that *R. obtusifolius* accumulates soluble oxalate to a greater degree than other *Rumex* species (Miyagi et al. 2010b).

In humans and livestock, exposure to oxalate irritates the gastrointestinal mucosa and leads to symptoms of calcium deficiency because it binds calcium in the body. Oxalate can also cause ureteral obstruction due to the development of calcium oxalate crystals in the kidneys, which can lead to death in severe cases (Dickie et al. 1978; Panciera et al. 1990; Reig et al. 1990). However, oxalate reportedly also functions as a useful bioactive substance in plants by mediating feeding defense (Franceschi and Nakata 2005) and tolerance to metal ions such as aluminum (Ma et al. 2001). Oxalate also serve as a source for the production of hydrogen peroxide, which functions as a signaling molecule in wounding (Le Deunff et al. 2004), aging (Davoine et al. 2001), and ripening (Huang et al. 2013) responses. Thus, oxalate accumulation may contribute to the environmental adaptation of *R. obtusifolius*.

Three oxalate synthesis pathways have been reported in plants: isocitrate, glycolate, and ascorbate pathways (Franceschi and Nakata 2005). Feeding of itaconate (methylene succinate) to *R. obtusifolius* grown in the dark was shown to cause the oxalate content in leaves to decline to less than one-tenth of the level in control plants, indicating that the isocitrate pathway plays a major role in oxalate synthesis in the leaves of *R. obtusifolius* (Miyagi et al. 2013b). Itaconate inhibits the activity of isocitrate lyase (ICL), a key enzyme that converts isocitrate to succinate and glyoxylate in the isocitrate pathway (Khan and Mcfadden 1979). However, ICL, the primary enzyme of the isocitrate pathway, is also reportedly expressed at night in other species (Nieri et al. 1997; Plancke et al. 2014). Moreover, an *ICL-*overexpressing line of rice exhibited a slightly increased oxalate content (Yu et al. 2010). No studies conducted in the presence of itaconate in bright light have been reported, however, so whether the isocitrate pathway functions as the primary pathway in bright light remains unclear.

The primary aim of the present study was to determine how differences in light and dark responsiveness affect oxalate synthesis in the leaves of *R. obtusifolius*. Primary metabolites (oxalate and related compounds) were measured using capillary electrophoresis-tandem mass spectrometry (CE-MS/MS) to evaluate changes in the oxalate synthesis pathway under inhibition of the isocitrate pathway.

## Materials and methods

### Plant materials

Seeds of *Rumex obtusifolius* L. were germinated on a petri dish. Each resulting seedling was transplanted into a Jiffy-7 pot (4 cm×4 cm×4.5 cm, Jiffy Products International AS, Norway). To avoid the effects of photoperiod and light stress on metabolite content, the plants were grown in a climate-controlled growth chamber under continuous low light (20 µmol m^−2^ s^−1^ at 22°C), as described by Miyagi et al. (2010b).

To avoid the movement of oxalate from old leaves to young leaves during itaconate treatment, all leaves of 2-month-old plants were removed, and the plants were watered with 10 mM itaconate solution under continuous light or dark conditions. After 3 weeks, “new leaves” (leaves newly emerged after all leaves had been removed; term as used in Miyagi et al. 2010a) were collected, immediately frozen in liquid nitrogen, and stored at −80°C.

### CE-MS/MS measurement of oxalate and other metabolites

Oxalate and other metabolites were extracted according to the methods described by Miyagi et al. (2010b), with minor modifications. The contents of 37 metabolites were measured using a CE-QQQ-MS system (CE: 7100; MS: G6420A; Agilent Technologies, Santa Clara, CA, USA) in the multi-reaction monitoring mode according to the methods described by Miyagi et al. (2020). Quantitative accuracy was determined by analysis of known concentrations of standard reference compounds using Agilent MassHunter Software (Agilent Technologies). The metabolite data obtained by CE-MS/MS analysis are shown in Supplementary Data 1.

### Statistical analysis

Multivariate analyses were performed using IBM SPSS, v27.0 (IBM, Armonk, NY, USA), according to the methods described by Miyagi et al. (2013a). Principal component analysis was based on correlation matrices (cases=plant samples, variables=metabolites). In hierarchical clustering analysis, the squared Euclidian distance and average linkage method (between groups) were developed. A heatmap was generated using Microsoft 365 Excel (Microsoft, Redmond, WA, USA). In these analyses, the content of each metabolite was normalized according to Z-score. Significance was assessed using Tukey’s HSD test, with significance defined as *p*<0.05.

## Results

**Figure 1:**
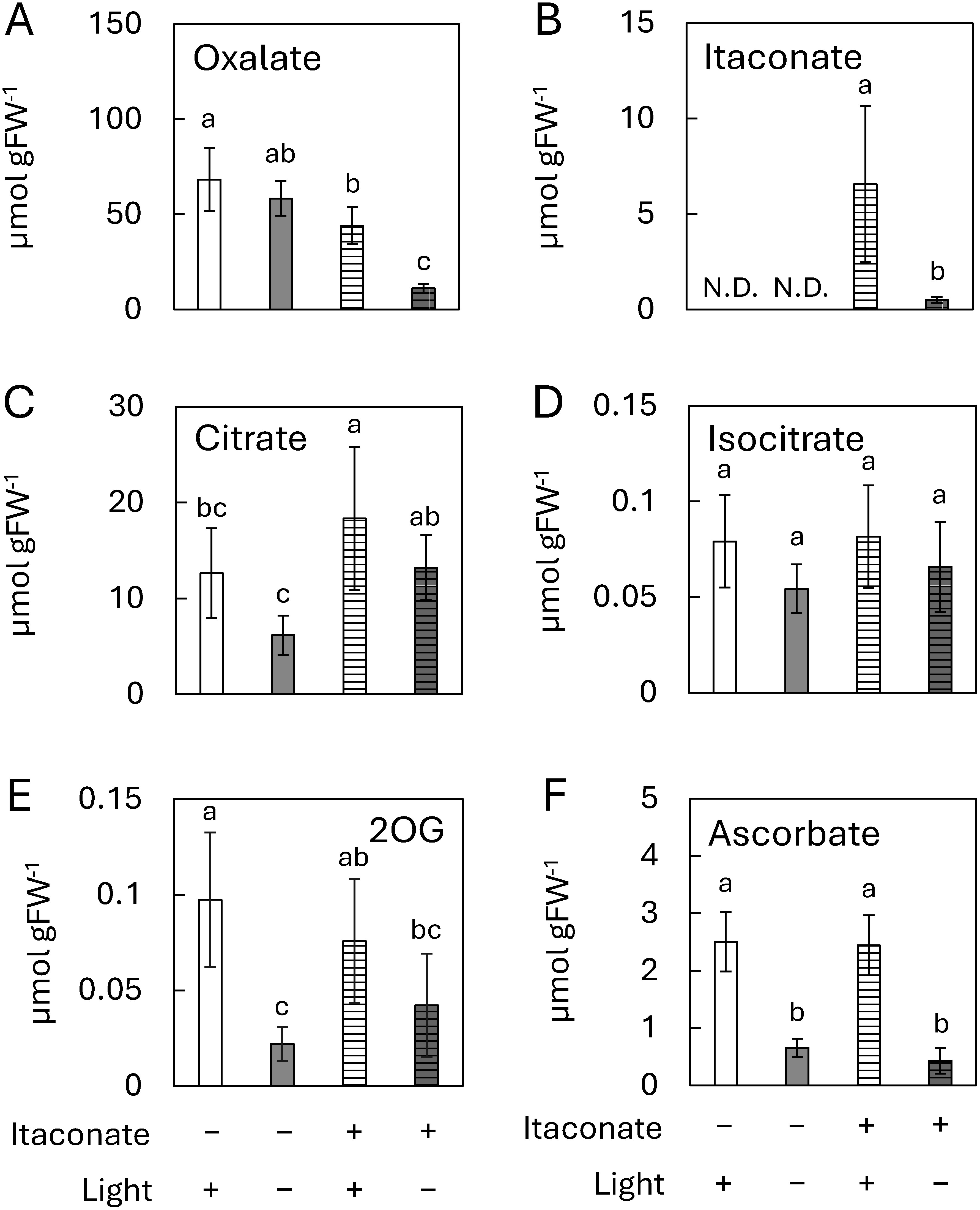
Figure 1. Effects of treatment with 10 mM itaconate solution and/or light irradiation for 3 weeks on oxalate accumulation (A), itaconate uptake (B), and contents of citrate (C), isocitrate (D), 2OG (E) and ascorbate (F) in new leaves of *R. obtusifolius* plants. Different letters indicate significant differences according to Tukey’s HSD test (*p*<0.05). *n*=6; bars, standard deviation.

To evaluate the effect of light on oxalate accumulation and the contents of other metabolites in leaves of *R. obtusifolius* treated with itaconate solution, we used CE-MS/MS to measure metabolite levels in new leaves of *R. obtusifolius* grown in the light with itaconate solution for 3 weeks. In the absence of itaconate solution, there was no significant difference in the oxalate content between leaves grown in the light and those grown in the dark (Figure 1A). However, the oxalate content was reduced in leaves of *R. obtusifolius* grown in the light with itaconate, while significantly decreased in leaves of plants grown in the dark with itaconate. No itaconate was detected in the leaves of *R. obtusifolius* not treated with itaconate solution (Figure 1B). In the leaves of *R. obtusifolius* treated with itaconate, approximately 13 times more itaconate accumulated in the leaves of plants grown in the light compared with the leaves of plants grown in the dark. With regard to metabolites that are directly involved in oxalate synthesis, citrate accumulated in leaves grown in the light with itaconate solution, whereas the citrate content was lowest in leaves grown in the dark without itaconate solution (Figure 1C). Differences in the light and itaconate treatment had no effect on isocitrate content (Figure 1D), and the pattern of 2OG content was similar to the pattern of citrate contents (Figure 1E). The ascorbate content increased in leaves grown in the light both with and without itaconate solution treatment (Figure 1F). Glycolate and glyoxylate, by contrast, were not detected in any leaves of *R. obtusifolius*.

**Figure 2:**
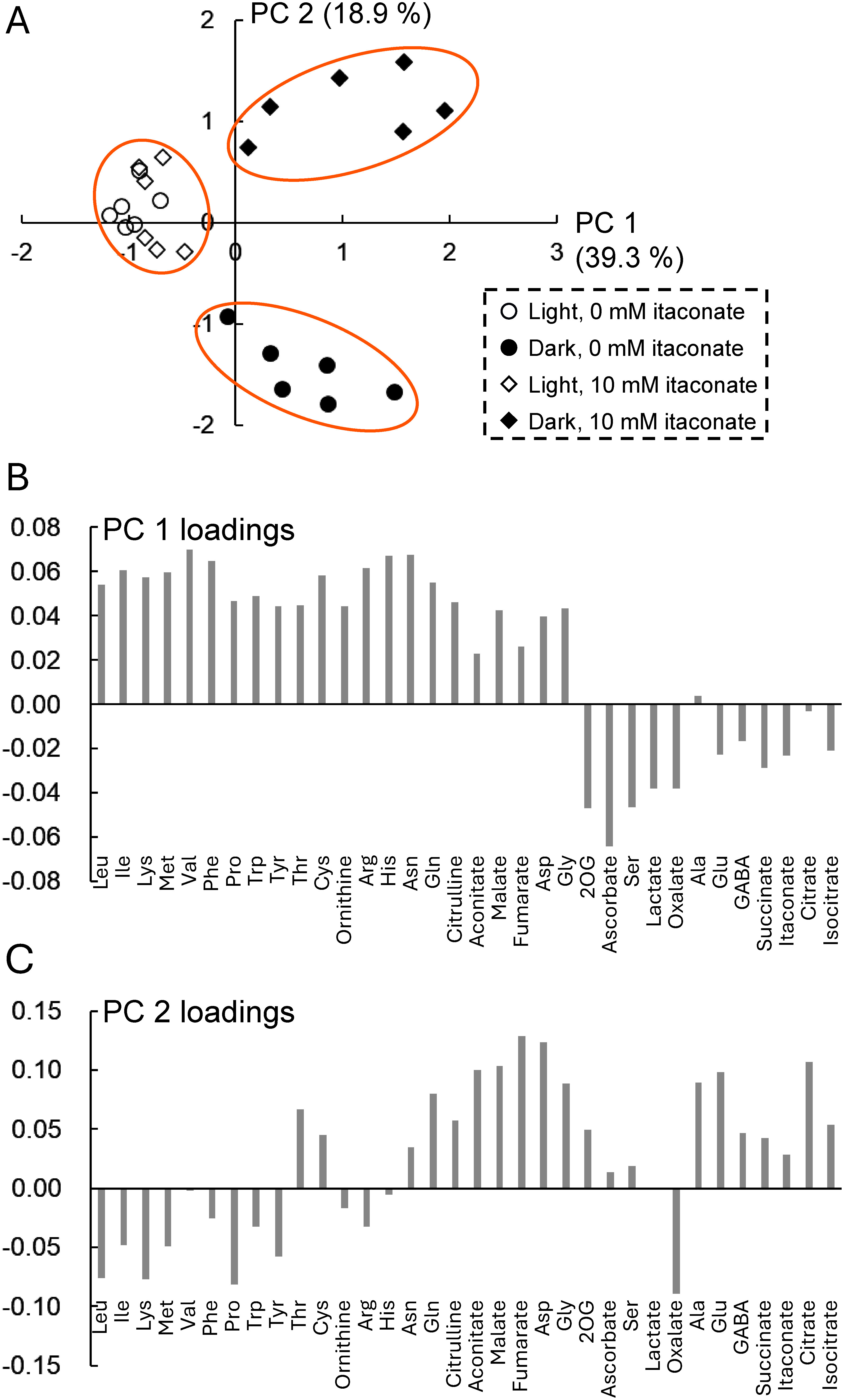
Figure 2. Principal component analysis based on the metabolite dataset of new leaves of *R. obtusifolius* plants grown in the light or dark with or without itaconate treatment for 3 weeks. (A) Principal component analysis scores are presented as combinations of two principal components (PC1 and PC2). Variance values (percent for PC1 and percent for PC2) are shown for each component. The loading score for each metabolite is shown for PC1 (B) and PC2 (C). Vertical axis shows the respective PC loading values (B and C).

We performed multivariate analyses of the metabolite dataset (34 metabolites excluding glycolate, glyoxylate, and homoserine because these metabolites were barely detectable in new leaves) obtained by CE-MS/MS to visualize the changes in metabolites associated with differences in itaconate treatment and/or light condition. Principal component analysis revealed three clusters. The first cluster consisted of plants grown in the light with or without itaconate, whereas the second cluster included plants grown in the dark without itaconate, and the third cluster consisted of plants grown in the dark with itaconate (Figure 2A). Plants grown in the light tended to cluster in the direction of PC1, whereas plants grown in the dark were distributed in the positive direction of PC1. For plants in the dark, those treated with itaconate were distributed in the positive direction of PC2, whereas plants grown without itaconate were distributed in the negative direction of PC2. Analysis of metabolite loading scores revealed that almost all amino acids as well as aconitate, malate, and fumarate contributed to the positive direction of PC1, although organic acids (particularly 2OG, ascorbate, lactate, and oxalate) and certain amino acids (e.g. serine) contributed to the negative direction of PC1 (Figure 2B). For PC2, organic acids (such as fumarate, citrate, malate, and aconitate), aspartate, alanine, glycine, glutamate, and glutamine contributed to the positive direction, although oxalate and some amino acids (proline, lysine, and leucine) contributed to the negative direction (Figure 2C).

**Figure 3:**
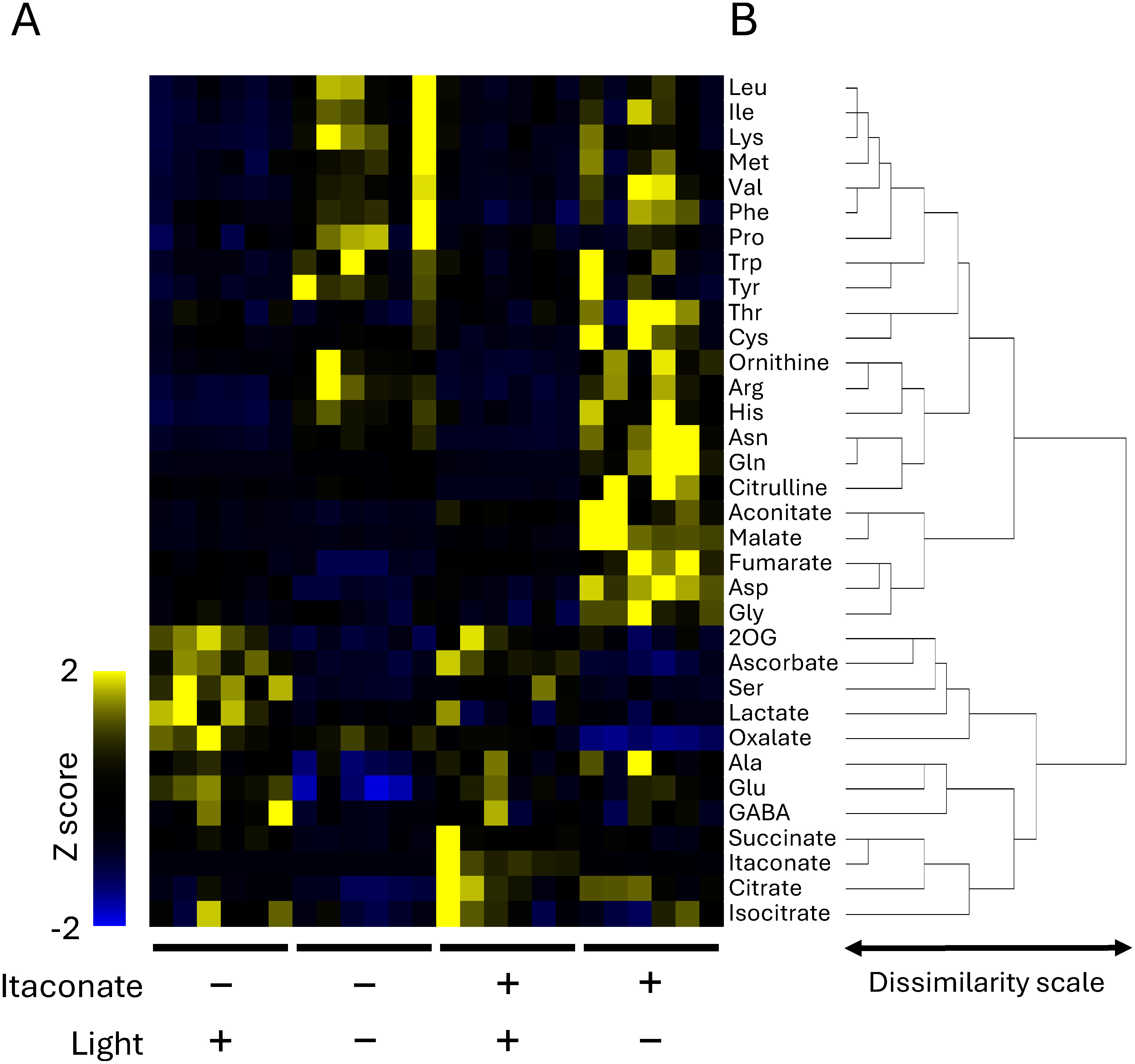
Figure 3. Heatmap (A) and dendrogram (B) generated using hierarchical clustering analysis based on the metabolite dataset for new leaves of *R. obtusifolius* plants grown in the light or dark for 3 weeks with and without itaconate treatment.

Hierarchical clustering analysis showed that metabolites could be divided primarily into two groups (Figure 3). Almost all amino acids and three organic acids (aconitate, malate, and fumarate) were included in the upper cluster, whereas the lower cluster consisted primarily of organic acids and four amino acids (serine, alanine glutamate, and γ-aminobutyrate). The contents of metabolites included in the upper cluster tended to increase in plants grown in the dark, whereas five metabolites (aconitate, malate, fumarate, aspartate, and glycine) increased only in plants grown in the dark with itaconate treatment. In the lower cluster, the contents of metabolites (except for oxalate) tended to be decreased in plants grown in the dark without itaconate treatment. Oxalate belonged to the same cluster as 2OG, ascorbate, serine, and lactate, whereas itaconate, succinate, citrate, and isocitrate formed a single cluster.

## Discussion

In the absence of itaconate treatment, there was no difference in oxalate content between the new leaves of *R. obtusifolius* plants grown in the light and those grown in the dark. The oxalate content of new leaves of *R. obtusifolius* grown in the dark was dramatically decreased by itaconate treatment. Itaconate was detected only in the new leaves of *R. obtusifolius* treated with itaconate. Leaf weight was unaffected by itaconate regardless of light exposure (Supplementary Figure S1), suggesting that the decrease in oxalate in new leaves of plants grown in the dark with itaconate solution was not due to itaconate-associated growth inhibition. A previous study reported that itaconate is an antagonistic inhibitor of ICL, which converts isocitrate to succinate and glyoxylate (Khan and Mcfadden 1979). These data suggest that itaconate-mediated inhibition of ICL activity would lead to a decrease in the oxalate content of *R. obtusifolius* leaves, consistent with our previous studies (Miyagi et al. 2010a, 2013b). Although citrate increased due to inhibition of ICL by itaconate in the dark, no change was observed in the isocitrate content. Isocitrate is also converted to 2OG and CO_2_ via decarboxylation by isocitrate dehydrogenase (IDH). Although itaconate had minimal impact on the 2OG content, the contents of amino acids such as Glu and Gln increased, as did the contents of succinate, fumarate, and malate. These findings suggest that isocitrate that is not utilized for oxalate synthesis due to itaconate-mediated ICL inhibition may be decarboxylated to 2OG by IDH and then converted to succinate and malate, or Glu and Gln through amino group addition, leading to their accumulation. However, the oxalate content of the leaves of *R. obtusifolius* plants grown in the light and treated with itaconate solution decreased slightly, whereas 10 times more itaconate accumulated in the new leaves of plants grown in the light compared with the new leaves of plants grown in the dark. The decrease in itaconate in the dark would likely be due to lower uptake rather than degradation. This is because the rates of leaf transpiration and root water absorption decrease in the dark due to the absence of photosynthesis, leading to reduced itaconate uptake. Another study reported that ICL activity is higher in the dark than the light (Nieri et al. 1997), which suggests that oxalate synthesis via the isocitrate pathway would occur only in the dark. In the present study, by contrast, the plants were grown under continuous light at an intensity (20 µmol photon m^−2^ s^−1^) lower than that in our previous studies (60 µmol photon m^−2^ s^−1^; Miyagi et al. 2010a, 2011, 2013b). Thus, we investigated the effect of light intensity on oxalate accumulation. Interestingly, under the high-intensity light condition (60 µmol photon m^−2^ s^−1^), the level of oxalate in new leaves treated with itaconate was the same as that of new leaves not treated with itaconate (Supplementary Figure S2). These results indicate that light intensity affects the rate at which the isocitrate pathway contributes to oxalate synthesis. Moreover, these data suggests that the ascorbate or glycolate pathway also contributes to oxalate synthesis in the light.

**Figure 4:**
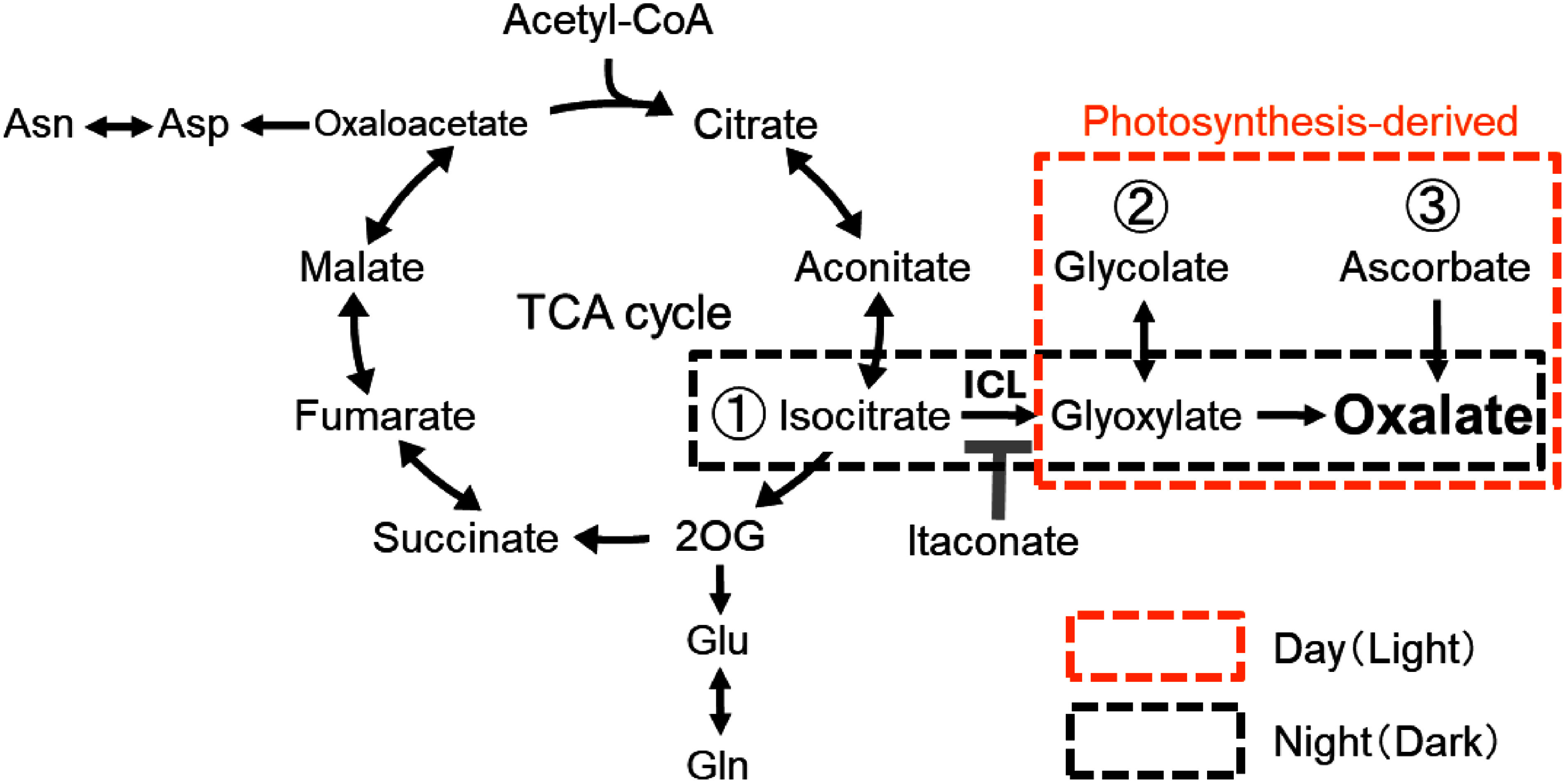
Figure 4. Putative oxalate synthesis pathway in oxalate-rich plants.

The ascorbate content of new leaves decreased in plants grown in the dark or treated with itaconate, as reported previously (Miyagi et al. 2010a, 2013b). Ascorbate, which is derived from mannose in plants, is synthesized in chloroplasts during exposure to light (Terai et al. 2020). Ascorbate plays a role in preventing oxidative damage caused by reactive oxygen species generated during photosynthesis. The observed decrease in ascorbate content in the dark could also be attributed to inactivation of ascorbate synthesis in the dark. By contrast, glycolate, which is derived from the photorespiration pathway, was not detected in either our previous studies (Miyagi et al. 2010a, 2013b) or the present study. The glycolate content would be extremely low because the light intensity in these experiments was lower than that of natural conditions in order to minimize the stress of continuous light exposure. Thus, oxalate would be synthesized primarily via the ascorbate pathway during the day, although it is synthesized via the isocitrate pathway during the night, as shown in Figure 4. The glycolate pathway may function under condition of high light intensity, such as those encountered in open fields. Further research is needed, however, to elucidate the effect of the glycolate pathway on oxalate accumulation under high-light conditions.

The results of the principal component analysis indicated that itaconate had a minimal effect on the metabolites of plants grown in the light, whereas metabolic changes centered on the oxalate synthesis pathway were induced by itaconate in the dark. In the hierarchical clustering analysis, itaconate, succinate, citrate and isocitrate clustered together, suggesting that changes in levels of citrate isocitrate, and succinate depend on the uptake of itaconate by new leaves. A strongly negative correlation between oxalate accumulation and itaconate content in the new leaves of plants grown in the dark was observed in our previous study (Miyagi et al. 2013b). Similar to the citrate content, the levels of other organic acids (such as malate) involved in the TCA cycle tended to increase after itaconate treatment of plants grown in the dark (Miyagi et al. 2013b). The levels of some amino acids (such as Glu, Asp, Gly and Ala) also increased following itaconate treatment in the dark. Increases in the levels of these metabolites would result from excess carbon produced due to the inhibition of oxalate synthesis by itaconate treatment, suggesting that inhibiting oxalate synthesis plays an important role not only in decreasing oxalate content but also in the production of other useful compounds.

In conclusion, we showed that the isocitrate pathway contributes only minimally to oxalate synthesis in the new leaves of *R. obtusifolius* grown in the light. This is the first report demonstrating that the contribution of oxalate synthesis pathways changes in the leaves of oxalate-rich plants. We also demonstrated that light intensity affects the contribution of oxalate synthesis pathways. However, why such changes in the contribution of oxalate synthesis pathways in response to changes in light intensity are needed in the leaves of *R. obtusifolius* remains unclear. Future studies should include genetic and transcriptomic analyses to elucidate the mechanisms of oxalate synthesis in greater detail.

## Abbreviations

2OG: 2-oxogluratate
CE-MS/MS: capillary electrophoresis-tandem mass spectrometry
GABA: γ-aminobutyrate
TCA cycle: tricarboxylic acid cycle

## Description of supplementary files

Supplementary Figure S1. Effect of treatment with 10 mM itaconate and/or light irradiation (20 µmol photon m^−2^ s^−1^) for 3 weeks on the fresh weight of new leaves of *R. obtusifolius* plants.

Supplementary Figure S2. Effects of treatment with 10 mM itaconate and/or light irradiation (60 µmol photon m^−2^ s^−1^) for 3 weeks on oxalate accumulation in new leaves of *R. obtusifolius* plants.

Supplementary Data 1. Metabolite contents of new leaves of *R. obtusifolius* plants grown in the light or dark for 3 weeks with or without itaconate treatment.
