# Supplementary material for: Oxalate synthesis pathways that contribute to oxalate accumulation in leaves of bitter dock (Rumex obtusifolius L.) differ between day and night
Source: Plant Biotechnol (Tokyo). 2026 Mar 25;43(1):89–94. doi: 10.5511/plantbiotechnology.25.1124a (PMC13170793; doi:10.5511/plantbiotechnology.25.1124a)
Supplement: Supplementary Data [file plantbiotechnology-43-1-25.1124a-s001.pdf]

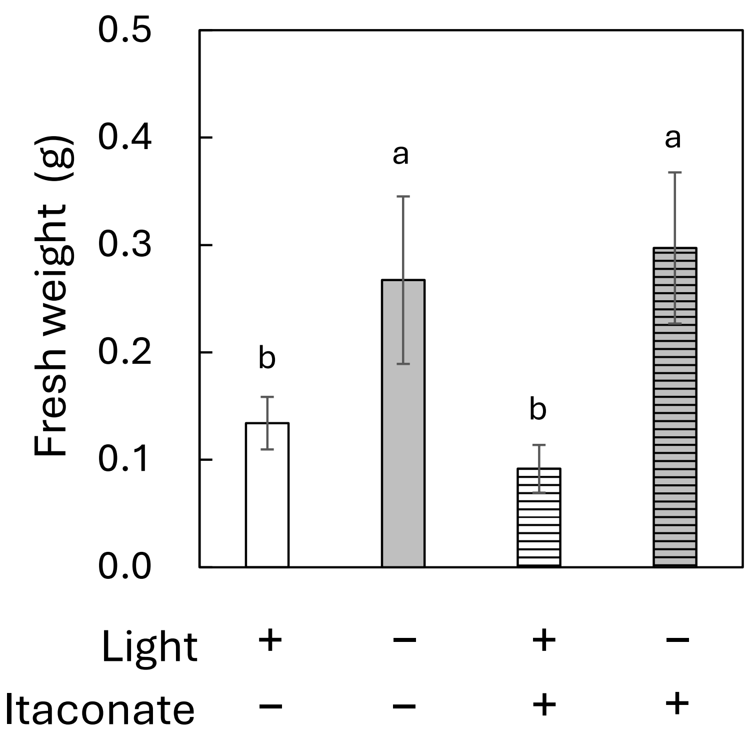

Supplementary Figure S1. Effect of treatment with 10 mM itaconate and/or light irradiation (20 μmol photon m^{-2} s^{-1}) for 3 weeks on the fresh weight of new leaves of *R. obtusifolius* plants. Different letters indicate significant differences according to Tukey's HSD test ($P < 0.05$). n = 6; bars, standard deviation.

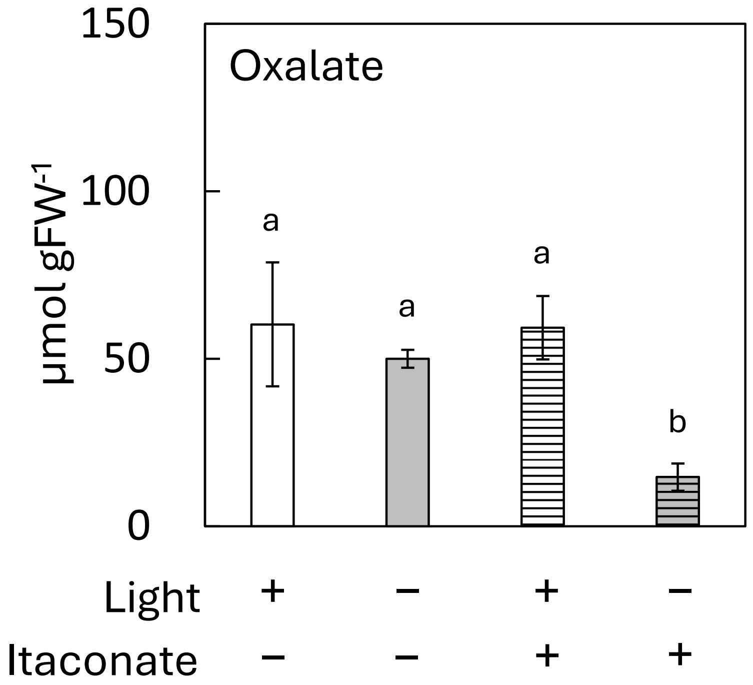

Supplementary Figure S2. Effects of treatment with 10 mM itaconate and/or light irradiation (60 μmol photon m^{-2} s^{-1}) for 3 weeks on oxalate accumulation in new leaves of *R. obtusifolius* plants. Different letters indicate significant differences according to Tukey's HSD test ($P < 0.05$). n = 6; bars, standard deviation.
